# Construction of nomogram model for risk of venous thromboembolism after spine surgery based on thromboelastography and coagulation indices

**DOI:** 10.3389/fmed.2024.1486190

**Published:** 2024-11-14

**Authors:** Yongtao He, Zhen Wang, Xiang Zheng, Xunmeng Zhang, Lianjin Guo

**Affiliations:** Department of Orthopedics, The Fourth Affiliated Hospital of Guangzhou Medical University, Guangzhou, China

**Keywords:** thromboelastogram, coagulation indices, venous thromboembolism, nomogram model, spine surgery

## Abstract

**Objective:**

To construct a nomogram model for the risk of venous thromboembolism after spinal surgery based on thromboelastography and coagulation indices and give relevant verification.

**Methods:**

Two hundred seventy-seven patients who underwent spinal surgery for spinal fractures admitted to our hospital were selected as the research subjects. According to whether venous thromboembolism occurred after surgery, they were divided into an occurrence group (confirmed by ultrasound or venography) of 34 cases and an absence group of 243 cases. The related materials, thromboelastograms and coagulation related indicators of the two groups were compared. The influencing factors of venous thromboembolism after spinal surgery were analyzed by univariate and multivariate regression models. Based on the influencing factors, the Nomogram model of venous thromboembolism after spinal surgery was established and its effectiveness was verified.

**Results:**

The proportion of patients whose age was ≥51 years old, the alpha Angle, the coagulation index (CI), the maximum thrombus amplitude (MA) and the levels of serum D-dimer (D–D), fibrinogen (FIB), fibrin degradation products (FDP), and thrombin-antithrombin complex (TAT) in the occurrence group were all significantly higher than those in the non-occurrence group. The clot formation time (*K*) and coagulation reaction time (*R*) were all lower than those in the non-occurrence group (*P* < 0.05). After Logistic multivariate analysis, alpha Angle, K, D-D, FDP, and TAT were all independent influencing factors of venous thromboembolism after spinal surgery (*P* < 0.05). Based on the independent influencing factors, the nomogram model of venous thromboembolism after spinal surgery was established, and the calibration curve was drawn. The consistency index was 0.838 (95% CI: 0.819–0.898), the goodness of fit test χ^2^ = 3.679, and *P* = 0.191 > 0.05. The calibration curve had a high degree of fit with the ideal curve. The clinical decision curve indicates that the net benefit of the prediction model is higher when the threshold probability is 0.1–0.9.

**Conclusion:**

A nomogram model based on alpha Angle, K, D-D, FDP, TAT and other independent influencing factors of venous thromboembolism in patients after spinal surgery has a high degree of fitting and high prediction value.

## 1 Introduction

Spinal surgery plays a key role in the treatment of spinal deformity, disc herniation, spinal fractures and other diseases. However, such operations often come with certain complication risks, and venous thromboembolism is a serious and possibly life-threatening one (1). Cutting, pulling and electrocautery of the tissues around the spine during the operation will lead to tissue damage, release a large number of tissue factors and inflammatory mediators, activate exogenous and endogenous clotting pathways, and maintain the blood in a hypercoagulable state. In addition, surgical trauma and postoperative pain will cause stress response of the body, resulting in increased secretion of hormones such as catecholamines and cortisol, which can promote platelet aggregation and the release of coagulation factors to increase the coagulation of blood (2, 3). The incidence of venous thromboembolism (including deep vein thrombosis and pulmonary embolism) after spinal surgery ranges from 0.2 to 13.6%, while the incidence of pulmonary embolism ranges from 0.03 to 2.4% (4). Currently, various methods such as physical and drug prevention are commonly used. However, some patients still form blood clots that affect blood circulation, cause limb swelling and discomfort, increase the risk of pulmonary embolism, affect postoperative recovery, increase medical costs, and lead to long-term complications. Therefore, after spinal surgery, close attention should be paid to changes in the patient's condition, and timely preventive measures and treatment methods should be taken to reduce the risk of thrombosis. Venous thromboembolism is an important complication of all large and time-dependent surgeries, including various orthopedic surgeries and spinal surgeries, which not only may lead to increased medical expenses, but also is an important cause of patient death (5). Anticoagulant therapy is a treatment of deep vein thrombosis, which plays an important role in controlling the development of thrombosis and reducing the risk of pulmonary embolism, but has no significant effect on the formed thrombosis (6, 7). Early prediction of the risk of venous thromboembolism after spinal surgery and timely intervention are of great significance to improve the prognosis and quality of life of patients. Venography is considered as the gold standard for venous thromboembolism, but it belongs to invasive surgery and causes great damage to patients (8). At present, there are a large number of reports on the related factors of venous thrombosis after orthopedic surgery, but the investigation may be incomplete, and there is no reliable prediction model. It has been found that hypercoagulable state of blood is an important risk factor for postoperative venous thromboembolism (9). Thromboelastography and coagulation indicators are commonly used methods for the detection of coagulation function. In this study, 277 patients undergoing spinal surgery in our hospital were taken as the research object. By analyzing the thromboelastogram and coagulation related indicators of patients, and based on this, nomogram model was established in order to diagnose the occurrence of venous thromboembolism after spinal surgery in the early stage and intervene in the treatment in time.

## 2 Data and methods

### 2.1 Clinical data

Two hundred seventy-seven patients who underwent spinal surgery for spinal fractures admitted to our orthopedic department were selected as the research subjects, and the selected time period was from July 2022 to May 2024 All patients underwent venous thromboembolism screening and were divided into an incidence group (diagnosed by ultrasound or venography) of 34 cases and a non incidence group of 243 cases based on whether deep vein thrombosis (DVT) occurred in the lower limbs 30 days after surgery. No thrombosis prevention strategy was implemented postoperatively. Inclusion criteria: ① Patients in the group with venous thromboembolism meet the diagnostic and treatment criteria for venous thromboembolism (10); ② Patients undergoing surgical treatment for spinal traumatic lesions, cervical disc herniation, and lumbar disc herniation; ③ Complete clinical data are available; ④ The patient was diagnosed with venous thromboembolism by imaging detection; ⑤ All the fractures were the first one, and there was no previous history of fracture. Exclusion criteria: ① Patients with preoperative thrombosis or a clear diagnosis of venous thromboembolism; ② Obvious abnormalities of liver and kidney function or heart function; ③ Patients who had taken coagulation-related drugs orally in the 2 months before participating in the study; ④ combined with infectious diseases or blood diseases; ⑤ During hospitalization, patients who did not undergo surgery, underwent non spinal surgery, underwent secondary surgical treatment, and had pelvic and lower limb fractures; ⑥ Surgical methods such as vertebral augmentation, bone cement filling, and spinal biopsy. ⑦ Patients with a history of concomitant venous thrombosis.

### 2.2 Clinical data collection

#### 2.2.1 General information

Including gender, fracture site (lumbar, thoracic, and cervical), diabetes history, hypertension history, and age (< 50 years, ≥51 years).

#### 2.2.2 Thrombus elastogram index detection

Perform thromboelastography testing on all patients after surgery, each venous blood sample was collected into a citrate-containing vacuum blood collection tube and analyzed within 2 h. All samples were maintained at room temperature, mixed gently by inverting the tube 5–10 times, and then run simultaneously on a thromboelastogram analyzer (Blood Technology, model TEG 5000). Add 20 μl of calcium chloride (CaCl_2_) to each cup and remove 360 μl of sample and start analysis immediately. Record relevant indicators of thromboelastography, including clotting time (*R* value): the time from the time when the blood sample was added into the test cup to the time when the amplitude of the curve opening reached 2 mm, reflecting the results of the comprehensive action of coagulation factors. Clot Forming Time (*K*-value): The time from the time starting point at which the R time was measured (that is, when clot formation began) to the time required for the trace amplitude to reach 20 mm, representing the rate of fibrin formation. Alpha Angle: It refers to the rate of formation of fibrin, which is positively correlated with the fibrinogen concentration. Maximum thrombus amplitude (MA): It reflects the maximum intensity of a blood clot, and is an indicator for evaluating the robustness of a blood clot.

#### 2.2.3 Coagulation index detection

After surgery, 4 ml of fasting venous blood was taken from the patient, and the upper clear fluid was collected. Within 30 min, coagulation analysis was performed using a blood analyzer (Shenzhen Shangdao Medical Technology Co., Ltd., model: DH36), Coagulation analysis, including activated partial prothrombin time (APTT) and thrombin time (TT), was performed within 30 min using a blood analyzer (Shenzhen Shangdao Medical Technology Co., Ltd., model: DH36). Plasma samples were taken from the subject. Reagent addition: APTT reagents containing contact activators, phospholipids, and calcium ions were added to the platelet-depleted plasma. Reaction conditions: The reaction was conducted at 37°C, with kaolin activation factor and cephalin replacing platelets to provide a catalytic surface for coagulation. Light irradiation: The sample was irradiated with light at a wavelength of 660 nm to observe the coagulation process. Recording time: from reagent addition until complete plasma coagulation, and the required time for recording was APTT value.

Enzyme-linked immunosorbent assay was used to detect the levels of D- dimer (D-D), fibrinogen (FIB), fibrin degradation products (FDP) and thrombin-antithrombin complex (TAT). Sample preparation: 3 ml fasting peripheral venous blood was collected in the morning of our patient, and plasma and serum were separated after centrifugation. Blood samples were collected using EDTA or heparin anticoagulation tubes, centrifuged at 3,000 rpm for 20 min or 1,000 g for 15 min at 4 C, and the supernatant was taken for immediate detection, or stored in a refrigerator at −20°C or −80°C but repeated freezing and thawing should be avoided. Solid phase plate preparation: 200 μl PBS buffer was added into each well of the solid phase plate for one-time plate washing, and then water was removed with absorbent paper. Sample addition and incubation: The samples to be tested, calibrators, and quality controls were added to the corresponding wells and fixed with phase plate for slight vibration to ensure that the solution completely covered the bottom of the wells. The phase plate was sealed and allowed to stand for 1 h while incubated with shaking at room temperature. Wash: Repeat the wash three times, adding 350 μ L of wash buffer each time to each well, and pipette the solution through a pipette or pipette and dry with a clean paper towel. Addition of enzyme conjugate working solution: 5 μL of enzyme conjugate working solution was added to each well site, and the samples were incubated at room temperature for 30 min after membrane sealing by shaking. The washing was repeated three times. Color reaction: 10 μl developer was added to each well and incubated for 15 min at room temperature in the dark. Termination of reaction: Then 5 μL stop solution was added into each well site, after the reaction is complete, measure the levels of D-D, FIB, FDP, and TAT using an enzyme-linked immunosorbent assay (ELISA) reader. Result reading: The optical density (OD) of each well was measured using a microplate reader, and the concentration of each index in the sample was calculated according to the standard curve.

The results were evaluated by at least two experienced hematology and coagulation specialists, who provided transfusion guidance based on the results.

### 2.3 Statistical methods

SPSS 23.0 was used for statistical analysis. The measurement data conforming to the normal distribution were shown as (?*x* ± *s*), and the comparison between groups was examined by independent sample *t*-test. Count data were expressed as [case (%)], and inter-group comparison was examined by χ^2^ test. Logistic regression analysis was performed with the occurrence of venous thromboembolism after spinal surgery as the dependent variable (1 = occurrence, 0 = non-occurrence). Nomogram model was established with R software, and Bootstrap self-sampling method was used for internal verification, and the calibration curve was drawn. The goodness-of-fit test was used to evaluate the fit of the model. The clinical net benefits of the nomogram model were analyzed by clinical decision curves. *P* < 0.05 was considered to be statistically significant.

## 3 Results

### 3.1 Comparison of clinical data between the two groups

There was no significant difference in the proportions of gender, fracture site, diabetes history, and hypertension history between the occurrence group and the non-occurrence group (*P* > 0.05). The proportion of patients with age ≥51 years old in the occurrence group was significantly higher than that in the non-occurrence group (*P* < 0.05, Table 1).

**Table 1:** Comparison of clinical data between the two groups [Cases (%)].

**Group**	**Occurrence group (*n* = 34)**	**Non-occurrence group (*n* = 243)**	**χ^2^**	***P***
**Age (years)**				
< 50	11 (32.35)	131 (53.91)	5.548	0.019
≥51	23 (67.65)	112 (46.09)		
**Gender (%)**				
Woman	18 (52.94)	114 (46.91)	0.434	0.510
Man	16 (47.06)	129 (53.09)		
**Fracture site (%)**				
Lumbar vertebra	16 (47.06)	123 (50.62)	0.534	0.766
Sternal vertebra	13 (38.24)	78 (32.10)		
Cervical vertebra	5 (14.71)	42 (17.28)		
**Diabetes history (%)**				
Be	5 (14.71)	34 (13.99)	0.013	0.911
No	29 (85.29)	209 (86.01)		
**History of hypertension (%)**				
Be	19 (55.88)	108 (44.44)	1.572	0.210
No	15 (44.12)	135 (55.56)		

χ^2^ represents the statistical test of count data between two groups.

### 3.2 Comparison of thromboelastogram indicators between the two groups

Alpha Angle, CI and MA levels in the occurrence group were higher than those in the non-occurrence group, and K and R levels were lower than those in the non-occurrence group (*P* < 0.05, Table 2).

**Table 2:** Comparison of thromboelastograms between the two groups ($\bar{x}\pm$ *s*).

**Group**	**Occurrence group (*n* = 34)**	**Non-occurrence group (*n* = 243)**	***t***	***P***
Alpha angle	80.99 ± 6.73	67.26 ± 5.84	12.594	< 0.001
CI	1.93 ± 0.69	1.08 ± 0.15	13.737	< 0.001
K (min)	0.73 ± 0.26	1.22 ± 0.37	7.463	< 0.001
MA (mm)	69.27 ± 6.29	52.39 ± 5.74	15.871	< 0.001
R (min)	3.12 ± 0.56	3.96 ± 0.59	7.822	< 0.001

t represents the statistical test of data between two groups.

### 3.3 Comparison of coagulation indicators between the two groups

The serum levels of D-D, FIB, FDP, and TAT in the Occurrence group were higher than those in the Non Occurrence group (*P* < 0.05). There was no significant difference in the levels of APTT and TT between the two group (*P* > 0.05, Table 3).

**Table 3:** Comparison of coagulation indexes between the two groups ($\bar{x}\pm$ *s*).

**Group**	**Occurrence group (*n* = 34)**	**Non-occurrence group (*n* = 243)**	***t***	***P***
APTT (s)	25.92 ± 5.41	24.85 ± 5.29	1.102	0.272
D-D (mg/L)	9.53 ± 2.30	5.49 ± 1.24	15.649	< 0.001
FIB (g/L)	4.19 ± 1.12	3.78 ± 1.09	2.047	0.042
FDP (μg/mL)	8.69 ± 2.17	1.57 ± 0.55	42.649	< 0.001
TAT (μg/L)	10.53 ± 3.17	4.74 ± 1.53	17.498	< 0.001
TT (s)	19.07 ± 5.30	18.47 ± 4.39	0.767	0.468

t represents the statistical test of data between two groups.

### 3.4 Single factor analysis of venous thromboembolism after spinal surgery

Logistic single factor analysis using the statistically significant indicators in Tables 1–3 showed that alpha Angle, CI, K, MA, D-D, FIB, FDP, and TAT were the influencing factors of venous thromboembolism after spinal surgery (*P* < 0.05, Table 4).

**Table 4:** Univariate analysis of venous thromboembolism after spinal surgery.

**Index**	**β**	**SE**	***Wald***	***P***	***OR***	***95% CI***
AGE	1.925	0.793	5.893	0.067	6.855	1.449–32.427
Alpha angle	1.113	0.258	18.610	< 0.001	3.043	1.835–5.048
CI	0.726	0.249	8.501	0.005	2.067	1.269–3.367
K	−1.069	0.266	16.151	< 0.001	0.343	0.204–0.578
MA	1.210	0.396	9.336	< 0.001	3.353	1.543–7.286
R	−0.862	0.355	5.896	0.066	0.422	0.211–0.847
D-D	2.083	0.464	20.153	< 0.001	8.029	3.235–19.925
FIB	0.825	0.327	6.365	0.047	2.282	1.202–4.332
FDP	0.875	0.298	8.622	0.003	2.399	1.338–4.302
TAT	3.258	1.123	8.417	0.004	25.997	2.878–234.862

β, regression coefficient; SE, standard error; WALD, calculate the ratio of regression coefficients to their standard errors; P, P-value; OR, ratio ratio.

### 3.5 Multivariate analysis of venous thromboembolism after spinal surgery

According to the statistically significant indicators in Table 4 and Logistic multivariate analysis, alpha Angle, K, D-D, FDP, and TAT were the independent influencing factors of venous thromboembolism after spinal surgery (*P* < 0.05, Table 5).

**Table 5:** Multivariate analysis of venous thromboembolism after spinal surgery.

**Index**	**β**	**SE**	***Wald***	***P***	**OR**	**95% CI**
Alpha angle	1.341	0.389	11.884	< 0.001	3.823	1.784–8.191
K	−1.147	0.422	7.388	0.035	0.318	0.139–1.377
D-D	2.190	0.663	10.911	< 0.001	8.935	2.438–32.753
FDP	0.787	0.233	11.409	< 0.001	2.197	1.391–3.496
TAT	3.859	1.043	13.689	< 0.001	47.418	6.141–366.134

β, regression coefficient; SE, standard error; WALD, calculate the ratio of regression coefficients to their standard errors; P, P-value; OR, ratio ratio.

### 3.6 Construction of nomogram model

**Figure 1:**
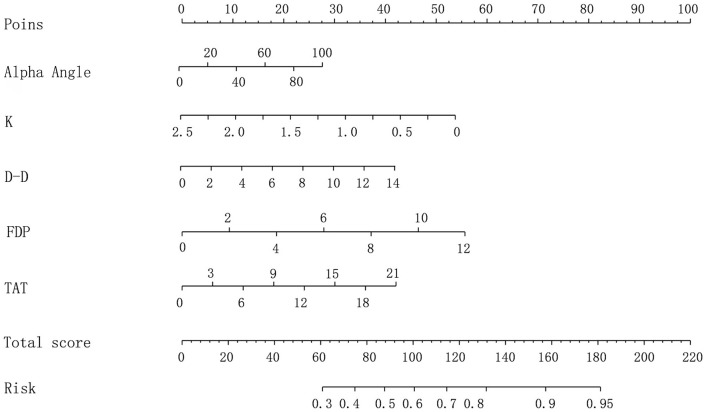
Nomogram risk prediction model.

Based on the independent influencing factors, a nomogram model for venous thromboembolism after spinal surgery was established, and the specific integral values of each independent risk factor were calculated according to the values of regression coefficients on the integral line at the top of the nomogram model (with the left endpoint corresponding to 0 point, see Figure 1).

### 3.7 Validation of nomogram model

**Figure 2:**
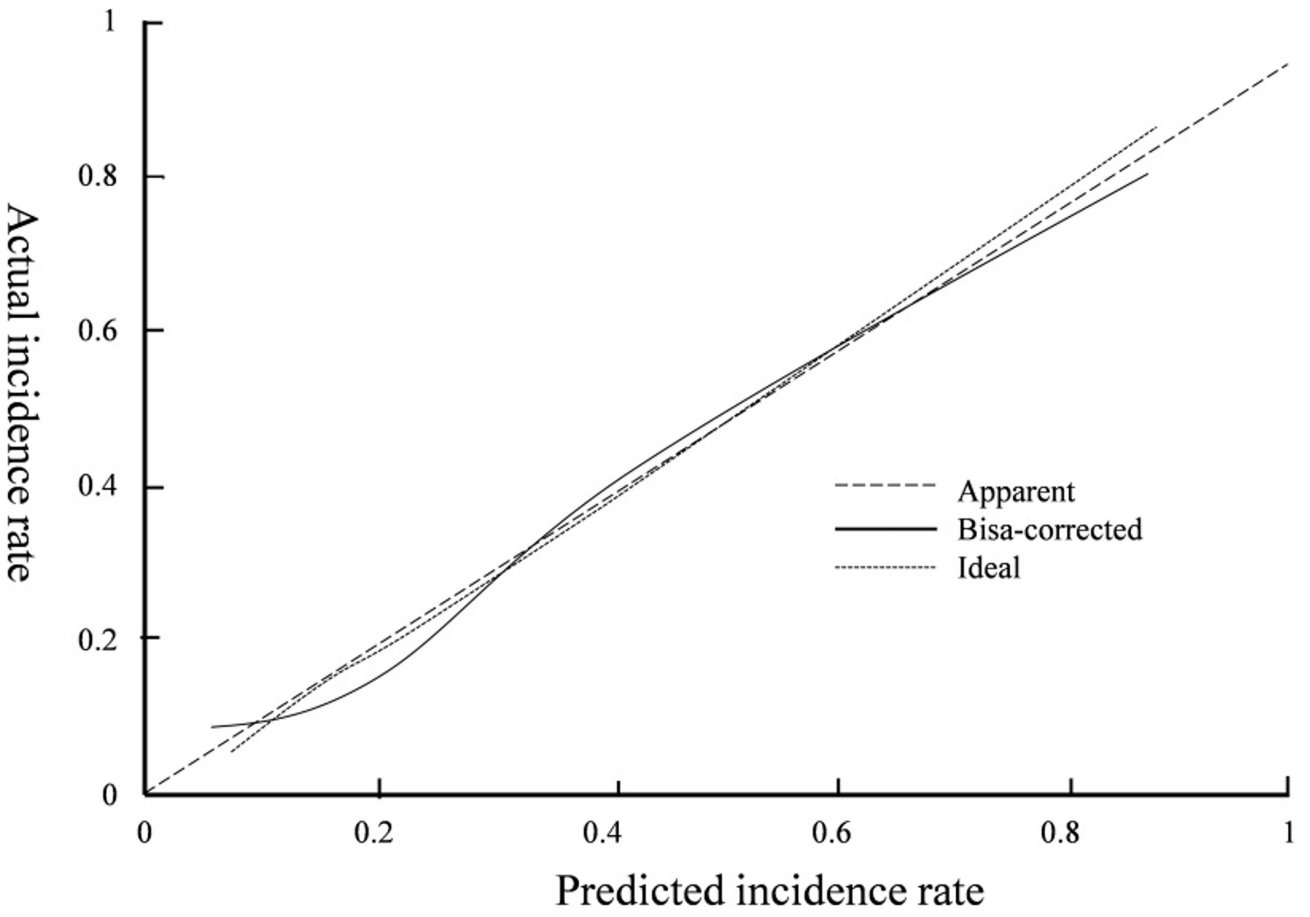
Calibration curve.

**Figure 3:**
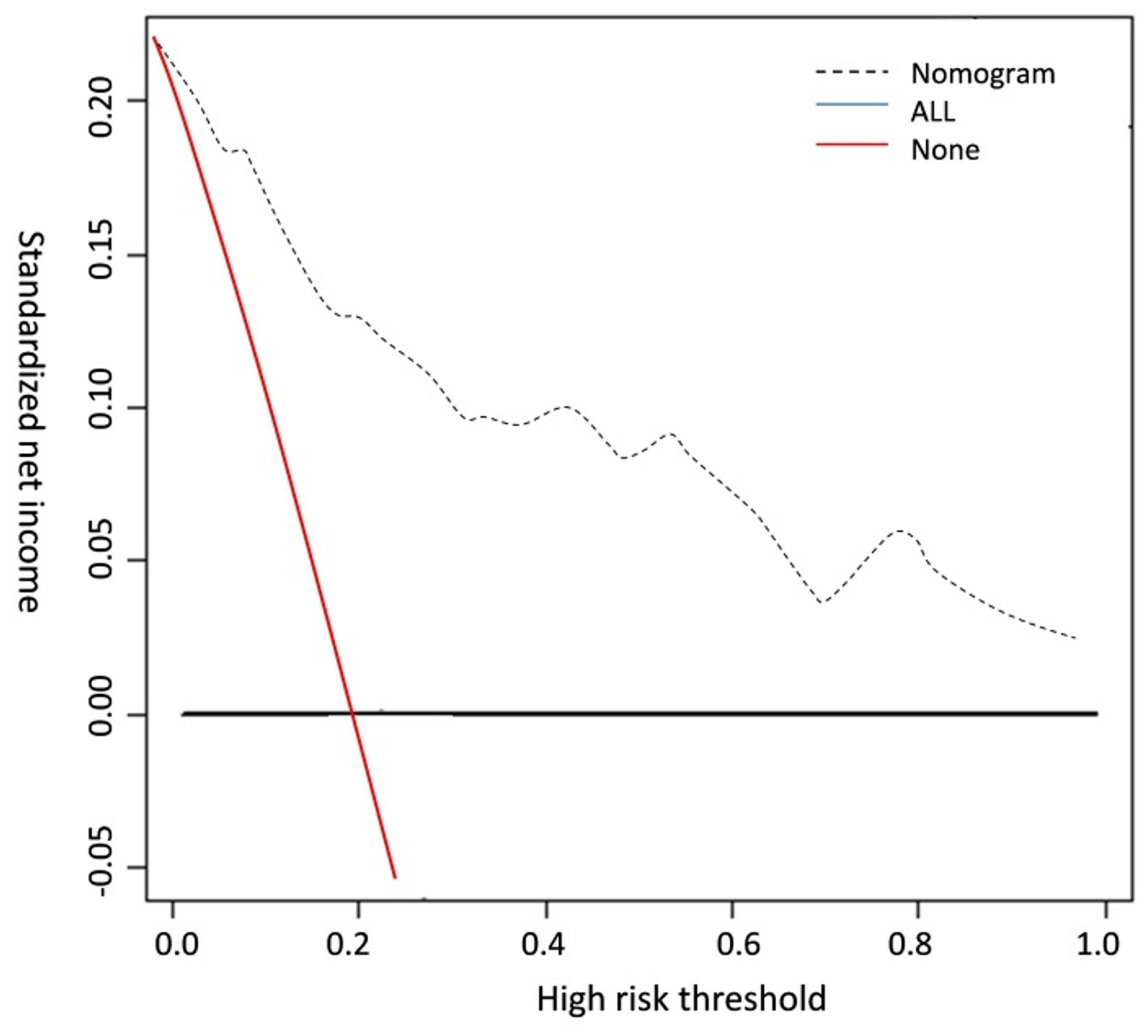
Clinical decision curve.

The occurrence of venous thromboembolism and the independent influencing factors were taken as state variables and test variables, respectively, to draw a calibration curve. The consistency index was 0.838 (95% CI: 0.819–0.898), and the goodness of fit test χ^2^ = 3.679, *P* = 0.191 > 0.05. The calibration curve had a high degree of fit with the ideal curve (see Figure 2). The clinical decision curve indicates that the net benefit of the prediction model is higher when the threshold probability is 0.1–0.9 (see Figure 3).

### 3.8 ROC curve analysis of D-D diagnosis of venous thromboembolism after spinal surgery and its clinical value

**Figure 4:**
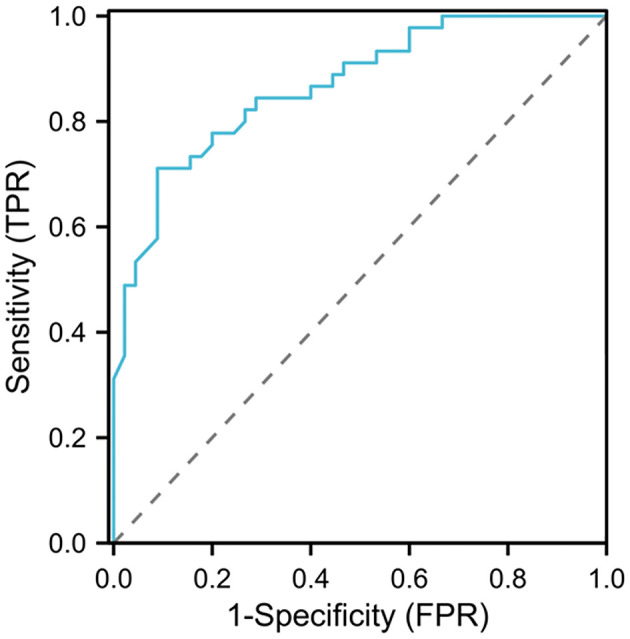
ROC curve analysis of the clinical value of D-D in diagnosing venous thromboembolism after spinal surgery.

The ROC curve analysis results showed that the area under the curve (AUC) for D-D diagnosis of venous thromboembolism after spinal surgery was 0.867, with a 95% CI of 0.795–0.939. When the D-Ddut off value was 6.87 mg/L and the maximum Yoden index was 0.622, the sensitivity was 71.11% and the specificity was 91.11% (see Figure 4).

## 4 Discussion

Venous thromboembolism caused by long-term immobilization is a life-threatening complication after all major orthopedic surgery. It is well-known that any type of surgery may produce a thrombogenic state due to stress and may increase the occurrence of postoperative thrombotic complications (11). According to statistics, the incidence of deep vein thrombosis after spinal surgery is about 0.3%−31% (12). In this study, we found that there were 34 cases of venous thromboembolism after operation among 277 patients undergoing spinal surgery, with the incidence rate of 12.27%, indicating that the incidence of venous thromboembolism after spinal surgery was high, which might be related to the decrease of muscle activity and slow blood flow velocity due to limb immobilization after operation. Therefore, it is necessary to understand the influencing factors of venous thromboembolism after spinal surgery and select the best prediction model for patients to predict the risk of venous thromboembolism at an early stage.

In this study, Logistic analysis showed that alpha Angle, K, D-D, FDP, and TAT were all independent influencing factors of venous thromboembolism after spinal surgery. Alpha Angle refers to the rate of clot formation, reflecting the function of fibrinogen and platelet aggregation ability. A larger alpha Angle means faster clot formation. *K* value represents the time of clot formation, reflecting prothrombin time and the function of some platelets. The short *K* value indicates rapid start of coagulation, and represents the effects of coagulation factors, PLT and FIB (13). The increase in alpha Angle and the decrease in *K* value reflect the over-activation of the coagulation system, resulting in the blood hypercoagulable state, which can both be used as indicators for evaluating the blood hypercoagulable state and fibrinolytic state. The thromboelastogram is based on the measurement of the viscoelastic properties of whole blood samples and various activities during coagulation, which can reflect the real-time tendency of thrombosis (14). Thromboelastography has been used in some studies to assess the risk of thrombosis in patients with colorectal cancer after surgery. It has been found that compared with conventional coagulation indicators, thromboelastography is more advantageous in assessing the risk of thrombosis in patients with colorectal cancer (15). It has been discovered in one study that the alpha Angle was significantly increased and the K was significantly decreased in the elderly patients with deep vein thrombosis of the lower extremities (16). Similar to the results of this study. With the increase of alpha Angle, the *K* value decreases, and the coagulation function is gradually enhanced. The elasticity of the blood clot is enhanced, and the fibrinolytic time is prolonged, thereby increasing the risk of venous thromboembolism (17). The formation of thromboembolism is often caused by coagulation disorders in patients, so coagulation indicators tend to become an auxiliary diagnostic tool for patients, help to understand the patient's coagulation function, and timely treatment (18). D-D was a degradation product of fibrin, and its increase suggested the existence of hypercoagulable state and hyperfibrinolysis in the body. FDP is involved in the formation and stability of thrombosis, and elevated level can increase blood viscosity. TAT, produced during the formation of fibrin polymer, is a complex formed by the combination of thrombin generation and antithrombin 1:1, which can indirectly reflect the amount of thrombin generated, serving as a marker for the initiation of the coagulation system and further predicting the formation of thrombus and the recurrence of thrombi at an early stage (19, 20). Studies have found that when myocardial infarction, deep vein thrombosis and other diseases occur, the levels of D-D, FDP and TAT are significantly increased, which can be used as useful screening parameters for thrombosis (21, 22). Similar to the results of this study. When the levels of D-D, FDP and TAT are increased, the coagulation function of the body is significantly increased, the activity of tissue plasminogen activator is decreased, and the degradation of fibrin is inhibited, thereby playing a role in promoting thrombosis (23). Tissue damage during spinal surgery will activate the coagulation system, leading to the release of coagulation factors and causing blood hypercoagulability. In addition, the stress caused by the operation causes the release of such hormones as catecholamines, which promotes platelet aggregation and increases blood coagulation. Under the stress state, the function of endothelial cells is damaged and the secreted anticoagulant substances are reduced, further promoting the hypercoagulable state (24). In patients with venous thromboembolism after spinal surgery, targeted treatment measures can be formulated according to the relevant risk factors to adjust the treatment regimen and improve the prognosis of the patient.

Nomogram is an intuitive graphical tool for rapid prediction of risk probability of clinical events, which converts the traditional regression model into a visual risk assessment for each patient, making it practical in clinical application. At present, nomogram has been widely used in the prediction of prognosis of cancer patients (25). In this study, nomogram was applied to the treatment of venous thromboembolism after spinal surgery. First, the more stringent variables were screened out by Logistic multivariate regression model and the nomogram model was established based on these variables. The results showed that the consistency index of the calibration curve of the nomogram model was 0.838 (95% CI: 0.819–0.898), and the model had a high degree of fit. Moreover, the prediction model had a high net benefit value when the value was 0.1–0.9, indicating that the model had a good prediction ability and clinical utility in the risk of venous thromboembolism after spinal surgery. The conclusions of this study have important guiding significance for clinical practice. A study has found that patients undergoing spinal surgery with elevated D-dimer levels, longer surgery times, and cervical spine surgery have a higher risk of developing VTE. Based on this, a column chart is constructed to provide theoretical basis for clinical doctors to prevent VTE (26). First of all, the preoperative detection of thromboelastograms and coagulation indicators can help clinicians to assess the risk of venous thromboembolism in patients after surgery, so as to develop targeted preventive measures. In addition, using the nomogram model, points corresponding to patient parameter values are identified, and based on their position on the graph, the patient's risk level is determined. According to the risk level, patients are classified into different categories such as low-risk, medium risk, and high-risk. Develop corresponding postoperative thrombosis prevention measures for patients with different risk categories. For example, high-risk patients may require more intensive anticoagulant therapy, while low-risk patients may only need to receive routine preventive measures. Compared with the Caprini scoring model, the performance of the new model constructed in this study has higher predictive accuracy and can more accurately identify high-risk patients. Therefore, it will have greater clinical value. In addition, if the new model is simpler and easier to use, it can reduce the workload of clinical doctors and improve evaluation efficiency, which will also be an important advantage. At the same time, if the new model covers more or more comprehensive risk factors for thrombosis, it will be able to more comprehensively assess the risk status of patients and provide more accurate prevention recommendations. This is of great significance for improving the prognosis of patients and reducing the medical burden.

## 5 Conclusion

In summary, the nomogram model was established based on the independent influencing factors of venous thromboembolism in patients after spinal surgery, such as alpha Angle, K, D-D, FDP, TAT, and the fitting degree was high, and the prediction value was high. However, for a single-center retrospective analysis in this study, there may be selection bias, which limits the universality of the conclusion, and there is a lack of external inflammation from different regions and ethnic groups. Hence, a large-scale and multi-center study can be conducted for further analysis in the later stage to verify the reliability of the conclusion.

## Data Availability

The raw data supporting the conclusions of this article will be made available by the authors, without undue reservation.
